# Performance evaluation of a direct chemiluminescence assay for 25-hydroxyvitamin D and its application in assessing vitamin D status among reproductive-age women in southeastern China

**DOI:** 10.1371/journal.pone.0356809

**Published:** 2026-09-15

**Authors:** Min Zhu, Tianhua Chen, Jiali Cao, Linhua Luo, Minchuan Lin, Haiying Huang, Weiwei Xu, Lihua Li, Hongbin Xie, Xiaoli Chen, Huiming Ye

**Affiliations:** 1 Department of Laboratory Medicine, Fujian Key Clinical Specialty of Laboratory Medicine, Women and Children’s Hospital, School of Medicine, Xiamen University, Xiamen, China; 2 Medical Laboratory of Xiamen Humanity Hospital Fujian Medical University, Xiamen, China; 3 Xiamen Innodx Biotech Co., Ltd, Xiamen, China; 4 Department of Health Management, Women and Children’s Hospital, School of Medicine, Xiamen University, Xiamen, China; Shiraz University of Medical Sciences, IRAN, ISLAMIC REPUBLIC OF

## Abstract

**Background:**

Vitamin D deficiency is a global public health concern, and women of reproductive age represent a particularly vulnerable population. Accurate measurement of serum 25-hydroxyvitamin D [25(OH)D] is therefore essential for reliable clinical assessment. This study aimed to evaluate the analytical performance of the Innodx vitamin D assay for 25(OH)D measurement and to investigate the prevalence of vitamin D deficiency among women of reproductive age in southeastern China.

**Methods:**

This cross-sectional study enrolled women of reproductive age (20–45 years) who sought medical services at Xiamen Maternal and Child Health Hospital in China between November 2023 and September 2025. Serum 25(OH)D levels were measured to determine vitamin D status using the Innodx vitamin D assay (Xiamen Innodx Biotech Co., Ltd., Xiamen, China). The analytical performance of the assay was validated according to Clinical and Laboratory Standards Institute (CLSI) guidelines, covering precision, accuracy, linearity, and interference. A method comparison was performed against liquid chromatography-tandem mass spectrometry (LC-MS/MS) and the Roche electrochemiluminescence assay. Based on the measured levels, vitamin D status was categorized as deficient (< 20 ng/mL), insufficient (≥20, < 30 ng/mL), or sufficient (≥ 30 ng/mL).

**Results:**

The intra-assay coefficients of variation (CV) were 3.18%, 2.09%, and 1.86% at three concentration levels (5, 25, and 100 ng/mL), while intermediate precision CVs were 4.34%, 3.64%, and 3.17%, respectively. Accuracy testing showed bias of 4.20% and 3.26% at 20 and 50 ng/mL. The assay demonstrated excellent linearity (R^2^ = 0.998) across the range of 4.0-150.0 ng/mL. No significant interference was observed from hemoglobin (100 mg/dL), bilirubin (40 mg/dL), triglycerides (400 mg/dL), or rheumatoid factor (600 IU/mL). In method comparison with LC-MS/MS, the Innodx vitamin D assay demonstrated strong concordance with a Pearson correlation coefficient of r = 0.970. When compared with the Roche vitamin D assay, the Innodx showed better correlation with LC-MS/MS. Among 495 women, the mean 25(OH)D concentration was 20.50 ± 7.88 ng/mL, with 88.9% (95% CI: 86.1%, 91.7%) having vitamin D deficiency or insufficiency. Women aged ≤ 25 years showed significantly lower levels (18.38 ± 6.26 ng/mL) compared to older groups (31–35 years: 23.31 ± 8.97 ng/mL; > 35 years: 23.72 ± 8.56 ng/mL) (*P* < 0.05).

**Conclusion:**

The Innodx vitamin D chemiluminescence assay demonstrated satisfactory analytical performance for clinical use. Our findings reveal a high prevalence of vitamin D deficiency among reproductive-age women in southeastern China, particularly in younger age groups, highlighting the need for public health interventions.

## Introduction

Vitamin D is a fat-soluble secosteroid essential for human health, playing critical roles in calcium homeostasis, bone metabolism, and immune function [1]. The serum concentration of 25(OH)D is recognized as the most reliable indicator of overall vitamin D status, reflecting both dietary intake and cutaneous synthesis [2]. Globally, vitamin D deficiency affects nearly one billion people and has emerged as a significant public health concern [3]. Furthermore, beyond its classical skeletal effects, vitamin D deficiency has been associated with increased risks of cardiovascular disease, diabetes, certain cancers, and autoimmune disorders [4].

25(OH)D serves as the major circulating metabolite of vitamin D. Owing to its abundance and stability, it is the established clinical biomarker for vitamin D status [2]. However, 25(OH)D is hydrophobic and thus poorly soluble in blood. About 90% of the total circulating 25(OH)D is bound to the vitamin D-binding protein (VDBP or DBP). The remaining 10% is bound to albumin, the main protein of human blood plasma. A tiny fraction representing approximately 0.04% of the total 25(OH)D concentration circulates as the free form [5]. The sum of all these fractions constitutes total 25(OH)D. Therefore, efficient release of 25(OH)D from its binding proteins is a critical analytical step.

Accurate measurement of serum 25(OH)D is essential for both clinical diagnosis and epidemiological research. The primary methodologies are liquid chromatography-tandem mass spectrometry (LC-MS/MS), which is regarded as the reference method, and various immunoassays. Although LC-MS/MS offers high specificity, its technical complexity and cost hinder routine clinical adoption. Consequently, chemiluminescence immunoassays have emerged as a practical alternative, offering superior automation, throughput, and accessibility [5]. However, methodological differences among assays can introduce result variability, thus underscoring the necessity for rigorous analytical validation.

The detection principle of 25(OH)D immunoassays addresses two critical technical challenges: first, releasing 25(OH)D from its binding proteins, VDBP or DBP and albumin; and second, enabling its specific immunodetection via competition between the released analyte and a labeled analogue. Currently, most commercial kits, including the Roche Elecsys Vitamin D Total assay and the Mindray VD-T assays (Mindray, Shenzhen, China), employ a pH-shift method that denatures binding proteins under acidic conditions to release 25(OH)D. In contrast, the Innodx assay utilizes an integrated pretreatment solution containing displacing agents, thereby streamlining the workflow by eliminating the separate pretreatment step.

Vitamin D receptors are widely expressed in female reproductive organs, implying a physiological link between vitamin D status and endocrinological functions [6]. The binding of vitamin D to these receptors regulates key processes such as follicular development, ovarian reserve, and endometrial receptivity. Moreover, experimental evidence shows that 1,25(OH)_2_D_3_ stimulates steroidogenesis in human ovarian tissue, increasing progesterone, estradiol, and estrone production by 13%, 9%, and 21%, respectively [7]. Clinically, vitamin D deficiency is implicated in the pathogenesis of polycystic ovary syndrome (PCOS), a major cause of female infertility [8]. Further supporting its clinical relevance, it has been proposed that vitamin D could serve as a biomarker of ovarian reserve and should be considered in the routine evaluation of infertility patients [9]. However, while animal studies consistently link vitamin D to infertility, findings from human studies remain inconclusive [6].

Given the rising prevalence of infertility, there is an urgent clinical need to assess vitamin D status in women of reproductive age. However, cutaneous vitamin D synthesis is influenced by multiple factors, including age, skin pigmentation, weather, clothing, sunlight exposure duration, and geographic location [10]. Therefore, even in regions with abundant sunshine such as southeastern coastal China, it remains essential to evaluate the prevalence of vitamin D deficiency among women of reproductive age in this region.

This study aimed to achieve two main goals. The first was to comprehensively evaluate the analytical performance of a domestic chemiluminescence immunoassay for serum 25(OH)D measurement, encompassing precision, accuracy, linearity, interference, and method comparison with LC-MS/MS and the Roche electrochemiluminescence assay. The second was to investigate the prevalence of vitamin D deficiency among reproductive-age women in southeastern China, a region with abundant sunshine yet potentially distinctive lifestyle factors that may influence vitamin D status.

## Materials and methods

### Population and sample

This cross-sectional study recruited fertile women who sought medical services at Xiamen Maternal and Child Health Hospital in China between November 2023 and September 2025. All participants were aged 20−45 years. Exclusion criteria included hepatic or renal dysfunction, thyroid disorders, metabolic bone diseases, or current use of medications that influence bone physiology. Serum samples were collected and analyzed using the Innodx Caris200 and Roche Cobas e 602 chemiluminescence platforms. Additionally, paired serum and lithium heparin (Li-heparin) plasma samples were collected from a subset of 55 participants. All samples were centrifuged and stored at −20 °C, with only one freeze-thaw cycle. This study was approved by the Ethics Committee of Xiamen Maternal and Child Health Hospital (KY-2021–013-H01, KY-2024–080-K02) and was performed following the Helsinki Declaration of 1964 and its later amendments. Informed consent was obtained from all subjects and medical records were deidentified for all personally identifiable information.

### Immunoassays and analyzer

The Caris200 fully automated analyzer (Xiamen Umic Medical Instruments Co., Ltd., Xiamen, China) and the 25(OH)D chemiluminescence immunoassay kit (Xiamen Innodx Biotechnology Co., Ltd., Xiamen, China) were employed for primary measurements. The assay utilizes a competitive chemiluminescence immunoassays principle for the quantitative detection of total 25(OH)D in human serum and plasma. The procedure was performed according to the manufacturer’s instructions with a total assay time of 30 minutes and a throughput of 200 tests per hour. In brief, the assay principle is as follows: 25(OH)D in human serum and plasma first binds to an acridinium ester-labeled anti-25(OH)D antibody, forming an “acridinium ester-25(OH)D antibody-25(OH)D” complex. Subsequently, magnetic microparticles coated with a 25(OH)D antigen analogue are added. These particles compete with the endogenous 25(OH)D in the sample for binding to the labeled antibody, forming an immunocomplex on the magnetic solid phase. Following magnetic separation and washing to remove unbound materials, the chemiluminescent signal is initiated by the sequential addition of pre-trigger and trigger solutions. The resulting signal is measured as relative light units (RLU). The RLU value measured by the analyzer is negatively correlated to the amount of 25(OH)D in the sample, and the 25(OH)D content in the sample is calculated from the standard curve. The Cobas e 602 (Roche Diagnostics Corporation, Germany) is a fully automated analyzer that performs competitive electrochemiluminescence immunoassays to detect vitamin D levels. Samples were tested according to the manufacturer’s instructions (Elecsys Vitamin D total, Roche Diagnostics.) with a total assay time of 27 minutes, with approximately 113 tests per hour. Cobas e 602 was used only in method-correlation studies.

### Liquid chromatography tandem mass spectrometry (LC-MS/MS)

A total of 100 clinical specimens were aliquoted and analyzed using LC-MS/MS as the reference method. Chromatographic separation was performed using an Agilent 1260 Infinity HPLC system, followed by detection with an Agilent Ultivo Triple Quadrupole Mass Spectrometer.

### Performance validation

The analytical performance of the Innodx immunoassay was comprehensively validated in accordance with both the “guiding principles of performance analysis of diagnostic reagents in vitro” by the Center for Medical Device Evaluation (CMDE) of NMPA and the Clinical and Laboratory Standards Institute (CLSI) documents. Key validation parameters encompassed precision, accuracy, linearity range, anti-interference capability, serum-plasma correlation, and method comparison against LC-MS/MS and the Roche immunoassay.

### Precision

System precision test for 25(OH)D assays was performed according to the People’s Republic of China WS/T492-2016 “Verification of Performance for Precision and Trueness of Quantitative Measurements in Clinical Laboratories” [11]. Intra-assay variability was determined by measuring samples of three 25(OH)D levels (5 ng/mL, 25 ng/mL, and 100 ng/mL). Each sample was measured 10 times on the same day. Intermediate precision was evaluated by performing one test on 20 separate days, with each test using samples of three 25(OH)D levels (5 ng/mL, 25 ng/mL, and 100 ng/mL). Intra-assay variability and intermediate precision were expressed as coefficients of variation (CV%), which were calculated as the standard deviation divided by the mean 25(OH)D concentration value. The National Health Commission’s inter-laboratory quality evaluation standard is used as the judgment. CV% of an intra-assay variability should not exceed 1/4 total allowable error (TEa), and CV% of an intermediate precision should not exceed 1/2 TEa.

### Accuracy

Accuracy verification was performed according to the health industry standard of the People’s Republic of China, WS/T 492–2016 “Verification of Performance for Precision and Trueness of Quantitative Measurements in Clinical Laboratories” [11]. Following the standard protocol, two concentrations of working calibrators (20 and 50 ng/mL) were analyzed in duplicate on five consecutive days, using the same assay procedure as for clinical samples. Bias was calculated for each measurement as: [(measured value – target value) / target value] × 100%. The mean bias across all measurements was then derived. Accuracy was considered acceptable if the mean bias was < 12.5% (≤ 1/2 of the total allowable error, TEa).

### Linearity range

Linearity was verified according to CNAS-GL037 “Guidance on the Verification of Quantitative Measurement Procedures used in the Clinical Chemistry” 6.4 Linear Interval Validation [12]. A high-concentration serum sample (H) (145.60 ng/mL) and a low-concentration serum sample (L) (4.75 ng/mL) were mixed to generate seven dilution levels (6H, 5H + L, 4H + 2L, 3H + 3L, 2H + 4L, H + 5L and 6L). Each level was analyzed in duplicate. Linear regression analysis was performed between the measured mean and the expected value to obtain y = bx + a and the coefficient of determination (R^2^) was calculated. The linear range was considered acceptable if the slope (b) was between 0.95 and 1.05 and R^2^ was ≥ 0.950.

### Anti-interference capability

Anti-interference studies were performed to determine whether 25(OH)D measurements were affected by endogenous substances such as hemoglobin (Sigma-Aldrich, Saint Louis, USA), triglycerides (TCI, Tokyo, Japan), rheumatoid factor (Seracare, Massachusetts, USA), or bilirubin (Aladdin Biochemical Technology, Shanghai, China). Serum samples with two 25(OH)D levels (11.43 ng/mL and 27.9 ng/mL) were spiked with hemoglobin, triglyceride, rheumatoid factor and bilirubin to assess potential interference. The final concentrations of potential interferents in the assay were as follows: hemoglobin at 0, 100, 500, and 1000 mg/dL; triglycerides at 0, 100, 200, and 400 mg/dL; rheumatoid factor at 0, 100, 300, and 600 IU/mL; and bilirubin at 0, 10, 20, and 40 mg/dL. Each sample was tested once. The interference rate was calculated as [(measured value – control value) / control value] × 100%, and the interference rate within ±5% was considered acceptable, indicating no significant interference.

### Method comparison

Method comparison involving the Innodx assay, Roche assay and LC-MS/MS was performed using 100 serum samples from routine patients. The results were evaluated using the Passing-Bablok regression analysis and Bland-Altman bias analysis. Clinical concordance between each immunoassay and LC-MS/MS was further evaluated by calculating overall concordance rates and Cohen’s kappa (κ) statistics with 95% confidence intervals (CIs).

### Serum and plasma sample comparison

Parallel analysis was performed on serum and Li-heparin plasma sample pairs (N = 55) from individual patients in a single analytical run, with each sample measured once.

### Detection and analysis of vitamin D levels in women of reproductive age

This study measured serum 25(OH)D concentrations in 495 women of reproductive age (20–45 years). Vitamin D status was classified according to the Endocrine Society Clinical Practice Guideline, deficiency (<20 ng/mL), insufficiency (≥20, < 30ng/mL), and sufficiency (≥30 ng/mL) [13]. To examine the association between age and vitamin D status, the cohort was stratified into four age groups: ≤ 25, 26–30, 31–35, and > 35 years.

### Statistical analysis

Statistical analysis was performed using GraphPad Prism (version 10.4.2) and MedCalc (version 23.3.7). Agreement between matched serum and Li-heparin plasma samples was evaluated by Passing-Bablok regression, with correlation expressed using Pearson’s coefficient (r) [14]. Method comparison was further assessed using Bland-Altman difference plots [15].Group differences were compared using one-way ANOVA, with statistical significance (*P* < 0.05) determined by Tukey’s HSD post hoc test.

## Results

### Precision

As summarized in Table 1, the intra-assay coefficients of variation (CV) for the three tested concentrations were 3.18%, 2.09%, and 1.86%, respectively, which met the requirement of not exceeding 1/4 TEa. As shown in Table 2, the intermediate precision CV values were 4.34%, 3.64%, and 3.17% for the three tested levels, respectively, which also met the requirement of not exceeding 1/2 TEa.

**Table 1:** Intra-assay variability of the 25(OH)D assay on the Caris200 analyzer.

	Mean (ng/mL)	SD	CV
**Level 1**	5.19	0.17	3.18%
**Level 2**	24.63	0.51	2.09%
**Level 3**	101.98	1.89	1.86%

**Table 2:** Intermediate precision of the 25(OH)D assay on the Caris200 analyzer.

	Mean (ng/mL)	SD	CV
**Level 1**	5.02	0.22	4.34%
**Level 2**	25.19	0.92	3.64%
**Level 3**	100.61	3.19	3.17%

### Accuracy

As shown in Table 3, the average bias of the 25(OH)D assay on the Caris200 analyzer at the two tested concentrations was 4.20% and 3.26%, respectively, which met the requirement of not exceeding 12.5% and also satisfied stricter thresholds of < 5%.

**Table 3:** Accuracy of the 25(OH)D assay on the Caris200 analyzer.

	Mean (ng/mL)	Target value (ng/mL)	Bias (%)
**Level 1**	20.84	20.0	4.20
**Level 2**	48.37	50.0	3.26

### Linearity range

**Fig 1:**
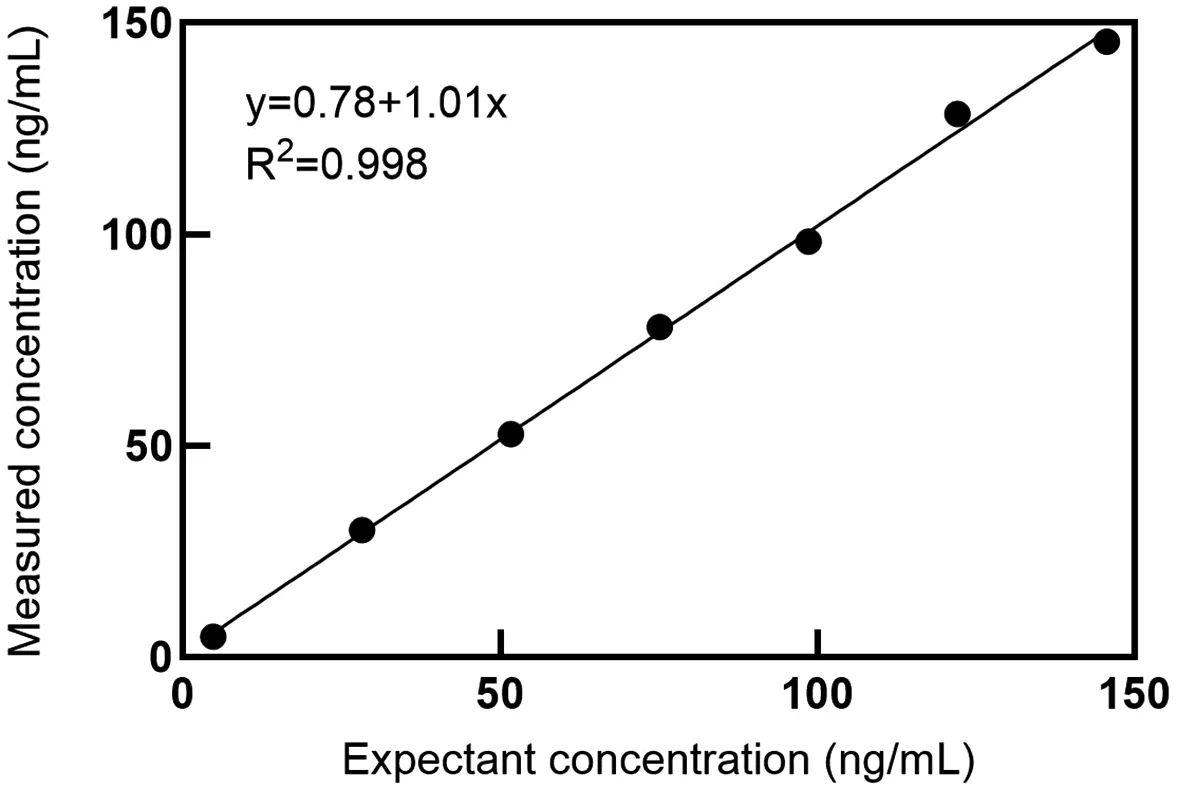
Linearity of the 25(OH)D calibration curve. The plot shows measured versus expected concentrations across the assay range, with linear regression analysis.

As shown in Fig 1, the assay demonstrated excellent linearity across the tested range. Linear regression of measured versus expected values yielded the equation y = 0.78 + 1.01x (R^2^ = 0.998), with R^2^ value consistently between 0.998 and 0.999.

### Anti-interference capability

To assess potential interference from endogenous substances on 25(OH)D measurements, serum samples with low concentration levels (11.43 ng/mL) and high (27.9 ng/mL) 25(OH)D concentrations were spiked with graded concentrations of hemoglobin, bilirubin, triglycerides, and rheumatoid factor (RF). As summarized in Table 4, the assay demonstrated acceptable robustness against clinically relevant concentrations of these interferents. Hemoglobin at 100 mg/dL (encompassing the typical clinical hemolysis range) showed interference rates below 5%; clinically significant bias was observed only with severe hemolysis (> 300 mg/dL). Bilirubin at concentrations up to 40 mg/dL yielded interference rates between −2.84% and 1.42%, all within the acceptable ±5% limit. Triglycerides up to 400 mg/dL produced interference rates ranging from −4.55% to 0.08%, meeting the non-interference criterion. For RF up to 300 IU/mL, the maximum interference was 3.10%, with all other values below 5%. In summary, at the tested thresholds—hemoglobin (100 mg/dL), bilirubin (40 mg/dL), triglycerides (400 mg/dL), and RF (600 IU/mL)—none of the endogenous substances produced clinically significant interference in the 25(OH)D assay.

**Table 4:** Interference assessment of the 25(OH)D assay on the Caris200 analyzer.

Substance	Concentration	Interference rate (%)	
		Level 1	Level 2
**Hemoglobin**	100 mg/dL	0.28	3.49
	500 mg/dL	6.31	4.24
	1000 mg/dL	7.79	6.76
**Bilirubin**	10 mg/dL	3.08	−3.64
	20 mg/dL	−4.33	−2.13
	40 mg/dL	−2.84	1.42
**Triglycerides**	100 mg/dL	−3.47	1.57
	200 mg/dL	2.68	−1.37
	400 mg/dL	−4.55	0.08
**Rheumatoid factor**	100 IU/mL	−0.17	0.04
	300 IU/mL	1.80	3.10
	600 IU/mL	0.09	−1.10

### Method comparison

**Fig 2:**
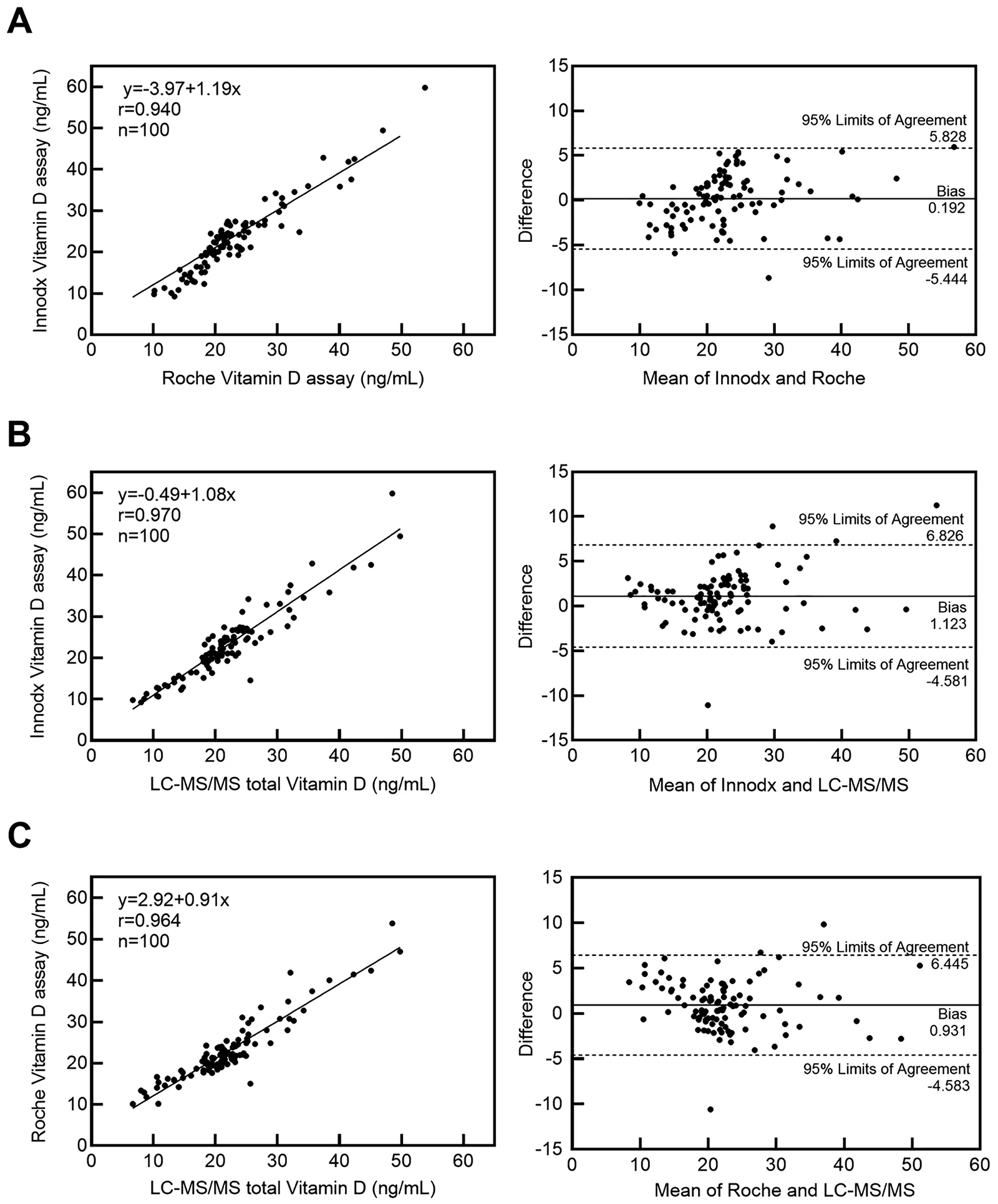
Method comparison of the Innodx vitamin D assay, LC-MS/MS and Roche vitamin D assay by Passing-Bablok regression and Bland-Altman plots. In Bland-Altman plot, absolute differences were calculated and plotted against the average of the two methods.

The analytical performance of the Innodx vitamin D assay was evaluated through method comparison with the Roche electrochemiluminescence assay and LC-MS/MS as the reference method (Fig 2). Initially, the two immunoassays demonstrated excellent mutual agreement (Fig 2A). Passing-Bablok regression yielded a slope of 1.19 (95% CI: 1.09 to 1.29) and an intercept of −3.97 (95% CI: −6.16 to −1.69) (r = 0.940). Bland-Altman analysis confirmed a minimal mean bias of +0.192 ng/mL, with 95% limits of agreement (LoA) from −5.444 to +5.828 ng/mL. Although the mean bias was small, the slope and intercept indicate proportional and constant differences between the assays. Therefore, the Innodx and Roche assays should not be considered directly interchangeable for individual patient monitoring. Subsequently, each immunoassay was compared individually to LC-MS/MS to evaluate accuracy (Fig 2B-C). Passing-Bablok regression revealed a strong correlation between the Innodx immunoassay and LC-MS/MS (slope = 1.08 [95% CI: 0.99 to 1.19], intercept = −0.49 [95% CI: −2.56 to 1.24], r = 0.970). Notably, the 95% confidence intervals for both parameters encompassed the ideal values of 1 and 0, indicating an absence of statistically significant systematic bias. In contrast, the Roche assay showed a slope of 0.91 [95% CI: 0.84 to 1.00] and an intercept of 2.92 [95% CI: 0.73 to 4.47] (r = 0.964), with the CIs excluding the ideal values, thus confirming both proportional and constant biases. Bland-Altman analysis against LC-MS/MS further quantified the differences, showing a mean bias of +1.123 ng/mL for Innodx and +0.931 ng/mL for Roche. The 95% LoA were similarly wide for both (approximately 11.4 ng/mL), highlighting a common limitation of immunoassays for tracking individual patients.

To evaluate the ultimate impact on clinical decision-making, clinical concordance was assessed by classifying samples (n = 100) according to LC-MS/MS results as deficient (< 20 ng/mL, n = 51), insufficient (≥20, < 30 ng/mL, n = 36), and sufficient (≥ 30 ng/mL, n = 13). Compared to this reference classification, the Innodx assay achieved an overall correct concordance rate of 82%, slightly higher than the 81% rate for the Roche assay (Table 5). Cohen’s kappa values were 0.700 (95% CI: 0.551-0.849) for Innodx and 0.686 (95% CI: 0.535-0.837) for Roche, which aligns with its marginally superior performance.

**Table 5:** Proportion of patient samples correctly classified against LC-MS/MS at different clinical decision thresholds.

LC-MS/MS 25(OH)D group	n	Concordant samples, n (%)	
		Innodx	Roche
**Deficient (< 20 ng/mL)**	51	44 (86.3)	41 (80.4)
**Insufficient (≥20, < 30 ng/mL)**	36	27 (75.0)	28 (77.8)
**Sufficient (≥ 30 ng/mL)**	13	11 (84.6)	12 (92.3)
**Total, n (%)**	100	82 (82.0)	81 (81.0)

Collectively, the data demonstrate that the Innodx immunoassay is highly concordant with an established commercial method. Furthermore, compared to the reference LC-MS/MS method, it exhibits a more favorable bias profile and achieves marginally superior clinical concordance, thereby supporting its reliability for serum 25(OH)D measurement.

### Serum and plasma

**Fig 3:**
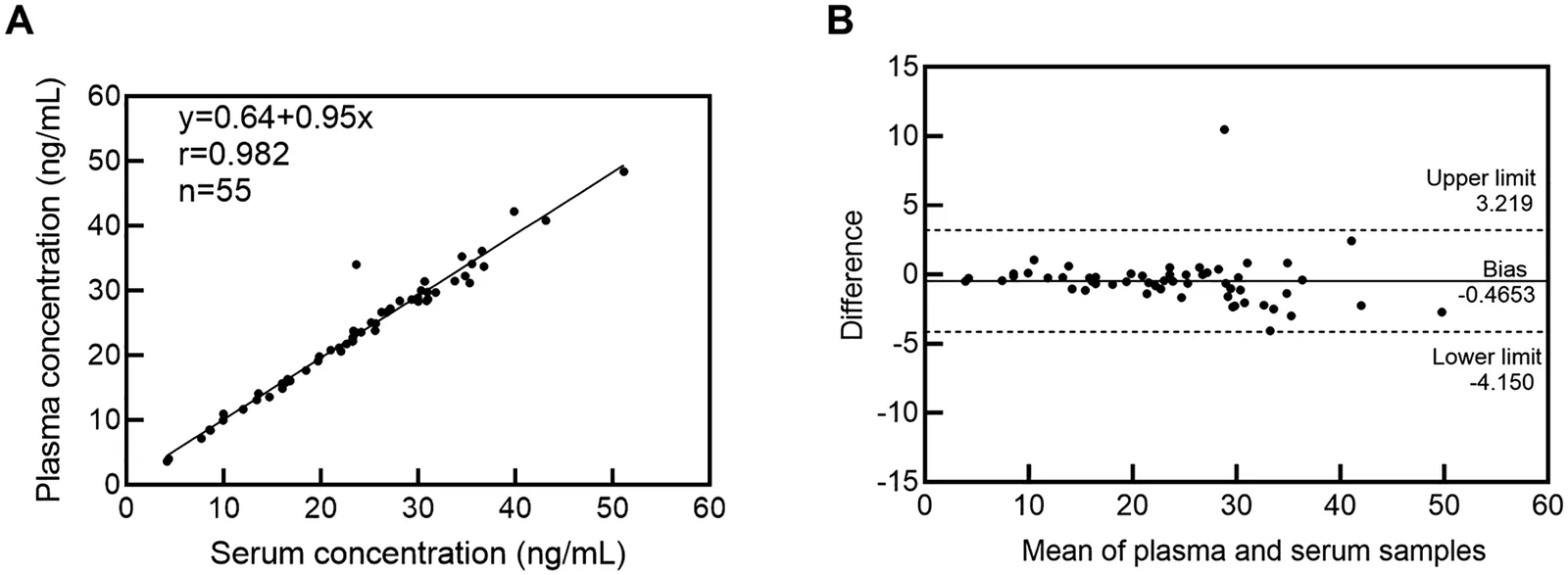
Correlation (A) and Bland-Altman difference (B) plots comparing serum and Li-heparin plasma 25(OH)D measurements.

The analytical agreement between serum and Li-heparin plasma samples for 25(OH)D measurements was evaluated in 55 matched pairs. Linear regression analysis yielded the equation of y = 0.64 + 0.95x (r = 0.982; Fig 3A). Bland-Altman analysis confirmed this systematic difference, showing a mean bias of −0.4653 ng/mL (Fig 3B). Collectively, these results demonstrate acceptable methodological consistency for 25(OH)D quantification within the measurement range of 4.0-50.0 ng/mL.

### Vitamin D level in women of reproductive age

The study included 495 women of reproductive age, with a mean age of 32.19 years. The mean serum 25(OH)D concentration was 20.50 ng/mL, below the sufficiency threshold of 30 ng/mL. Consequently, 88.90% of participants were classified as vitamin D deficient or insufficient (< 30 ng/mL) (Table 6). The mean 25(OH)D concentrations across four age groups were 18.38 ± 6.26, 21.20 ± 8.85, 23.31 ± 8.97, and 23.72 ± 8.56 ng/mL, respectively (Table 6). Notably, the mean 25(OH)D level in the youngest age group (≤ 25 years) was less than 20 ng/mL, corresponding to vitamin D deficiency.

**Table 6:** Age group distribution of serum 25(OH)D levels in women of reproductive age.

Age group		25(OH)D (ng/mL)			Total	Mean ±SD
		< 20	≥20, < 30	≥30		
≤**25**	Number	43	29	2	74	18.38 ± 6.26
	%	8.70%	5.90%	0.40%	14.90%	
**26-30**	Number	73	51	13	137	21.20 ± 8.85
	%	14.70%	10.30%	2.60%	27.70%	
**31-35**	Number	68	76	22	166	23.31 ± 8.97
	%	13.70%	15.40%	4.40%	33.50%	
**> 35**	Number	47	53	18	118	23.72 ± 8.56
	%	9.50%	10.70%	3.60%	23.80%	
**Total**	Number	231	209	55	495	20.50 ± 7.88
	%	46.70%	42.20%	11.10%	100.00%	

## Discussion

25(OH)D is an important metabolite in the human body that represents the main storage form of vitamin D. It is usually detected in the blood and is an indicator of total vitamin D levels in the body [2]. The domestic chemiluminescence platform (Innodx) has developed a new assay for 25(OH)D measurement. This study evaluated the precision, accuracy, linearity, and anti-interference ability of the chemiluminescence method for detecting 25(OH)D in line with the performance evaluation requirements of CNAS-GL037 “Guidance on the Verification of Quantitative Measurement Procedures used in the Clinical Chemistry” [12]. The results showed that the performance of 25(OH)D meets clinical requirements. The average bias of accuracy for 25(OH)D met the 1/2 TEa requirement and also satisfied stricter thresholds of < 5%.; the CV of intra-assay variability and intermediate precision for 25(OH)D were below 5% and 10%, respectively, which meets the precision requirements stated in the national standard. Moreover, the linearity analysis revealed excellent correlation indexes in its linear range (4.0 ng/mL to 150.0 ng/mL). The Innodx assay correlated well with LC-MS/MS (r = 0.970) and showed consistency with a leading international assay (r = 0.965), supporting its robust analytical performance for clinical application.

Although the Innodx vitamin D assay and the Roche assay showed a strong linear relationship and yielded broadly concordant classification of vitamin D status at clinically relevant thresholds, their quantitative 25(OH)D results were not identical. The deviation of the Passing–Bablok slope from unity and the non-zero intercept indicate proportional and constant differences between the two analytical platforms. Therefore, despite their comparable performance for population-level screening and vitamin D status classification, the Innodx and Roche assays should not be used interchangeably for longitudinal monitoring of individual patients, dose-adjustment decisions, or direct comparison of quantitative 25(OH)D concentrations across studies or clinical settings using different analytical platforms.

One advantage of the Innodx vitamin D assay is that the linear range is broader than that of the Elecsys vitamin D total III assay (3.0 ng/mL to 120.0 ng/mL). Thanks to the wide linear range of the Innodx chemiluminescence platform, very few clinical samples require dilution for proper 25(OH)D measurement. Moreover, the Innodx assay utilizes only three reagent components and requires no pretreatment. In contrast, the Roche assay employs five components, including two pretreatment reagents to release 25(OH)D from the VDBP, which occupy additional analyzer,positions and thereby limit overall testing throughput.

Our study found that 88.9% of 495 women of reproductive age have a deficiency or insufficiency of 25(OH)D. The mean 25(OH)D concentration were 18.38 ± 6.26 ng/mL in those aged ≤ 25 years and 21.20 ± 8.85 ng/mL in those aged 26−30 years. These results were nearly consist with previous studies in northern China [16,17], The high prevalence of vitamin D insufficiency or deficiency may be partly explained by the daily application of cosmetics containing sun-protection ingredients by women, which probably prevents the skin from exposure to ultraviolet-B (UV-B) light to trigger *in situ* biosynthesis of VD_3_. Vitamin D has been found to have an important biological role in female reproduction. There are vitamin D receptors in the ovaries, uterus, endometrium and placenta [18]. Vitamin D may contribute to embryonic implantation, placentation and pregnancy success [6]. Previous studies showed that women with 25(OH)D above 20 ng/mL had a higher possibility of pregnancy than those with 25(OH)D below 20 ng/mL. This correlation has also been observed in studies conducted in the US, where 25(OH)D status have been associated positively with a probability of pregnancy [6]. Moreover, a strong association exists between maternal and childhood vitamin D insufficiency [19–22]. Women commonly remain unaware of their vitamin D inadequacy as it often causes no perceptible discomfort. Some studies showed that adequate vitamin D status might increase the probability of pregnancy and live births in women during infertility treatments, such as in vitro fertilization (IVF) [23]. Vitamin D supplementation was found to alter gene expression in granulosa cells in women undergoing IVF. It has been reported that the effect of vitamin D on infertility may also be by increasing the level of interleukin-6 (IL-6) which is one of the pro-inflammatory cytokines. In a study, it was found that women with both low serum vitamin D levels and high IL-6 levels had a higher risk of infertility due to tubal factors. It has also been pronounced that inflammatory factors such as IL-6 show a significant correlation in individuals with endometriosis complicated with infertility [24]. Another study has demonstrated that 1α,25-(OH)_2_D_3_ promotes calcium transport in the placenta, stimulates placenta lactogen expression, and regulates HOXA10 expression in human endometrial stroma cells [7]. HOXA10 plays an important role in promoting the development of the uterus and endometrial which allows good uterine receptivity to implantation [7]. Previously, it has been proposed that a potential role of vitamin D in ovarian follicular development and function from primordial follicle activation to ovulation to generate mature oocytes [25]. The specific mechanisms and correlation between vitamin D levels and assisted reproduction treatment outcomes require further investigation.

A limitation of this study is the relatively small sample size for specific analytical comparisons. Although the overall cohort was substantial (N = 495), the subsets used for method validation (e.g., LC-MS/MS vs. immunoassay) were more limited. Therefore, findings related to method agreement warrant cautious interpretation and should be confirmed in larger multicenter studies covering broader 25(OH)D concentration distributions. Additional limitations include the lack of detailed data on season, body mass index, sun exposure, dietary intake, supplement use, clothing habits, and sunscreen use, all of which may influence vitamin D status. Currently, no evidence-based guidelines exist for vitamin D supplementation specifically in women of reproductive age. While the Endocrine Society Clinical Practice Guidelines [12] provide general recommendations for adults (daily intake: 1,500−2,000 IU vitamin D_3_; upper limit: 10,000 IU/day), prospective studies are needed to determine the optimal dosage and target serum 25(OH)D levels for this population. Given the high prevalence of vitamin D deficiency observed and its potential implications for reproductive health, achieving vitamin D sufficiency represents an important public health objective.

## Conclusion

In conclusion, the Innodx chemiluminescence immunoassay provides satisfactory analytical performance for 25(OH)D measurement, with robust agreement against LC-MS/MS. Its application in a clinical cohort from southeastern China validated the assay’s ability to identify a high prevalence of vitamin D insufficiency, a finding that was most pronounced among younger women. These results confirm its utility for routine screening and reinforce the need for precise vitamin D assessment. Further research should investigate its implementation in broader clinical contexts.

## Supporting information

S1 Data Data. (XLSX)
