# Supplementary material for: Performance evaluation of a direct chemiluminescence assay for 25-hydroxyvitamin D and its application in assessing vitamin D status among reproductive-age women in southeastern China
Source: PLoS One. 2026 Sep 15;21(9):e0356809. doi: 10.1371/journal.pone.0356809 (PMC13577506; doi:10.1371/journal.pone.0356809)
Supplement: Supplementary file 1 [file pone.0356809.s002.docx]

Dear Editor,

We thank the reviewers and you for the opportunity to revise and resubmit our work as well as for their comments that helped us considerably improve the content and presentation of the paper. We have performed a very thorough revision of the paper, resulting in a significantly improved manuscript. We have also addressed reviewers’ comments line by line. All revisions in the revised manuscript are tracked using Microsoft Word’s Track Changes function.

We have provided our explanations and responses after the comments below.

Sincerely,

When submitting your revision, we need you to address these additional requirements.

1. Please ensure that your manuscript meets PLOS ONE's style requirements, including those for file naming. The PLOS ONE style templates can be found at

https://journals.plos.org/plosone/s/file?id=wjVg/PLOSOne_formatting_sample_main_body.pdf and

https://journals.plos.org/plosone/s/file?id=ba62/PLOSOne_formatting_sample_title_authors_affiliations.pdf.

Response: The manuscript has been thoroughly revised to fully comply with the PLOS ONE formatting requirements, including file names.

2. Thank you for stating in your Funding Statement:

“This work was financially supported by the Xiamen Major Science and Technology Program (Milestone Project, 3502Z20254028, Huiming Ye), the Medical and Industrial Integration Guidance Project of Xiamen (3502Z20244ZD2043, Min Zhu), the Fujian Provincial Natural Science Foundation of China (2023J05266, Jiali Cao). “

Please provide an amended statement that declares *all* the funding or sources of support (whether external or internal to your organization) received during this study, as detailed online in our guide for authors at http://journals.plos.org/plosone/s/submit-now. Please also include the statement “There was no additional external funding received for this study.” in your updated Funding Statement.

Please include your amended Funding Statement within your cover letter. We will change the online submission form on your behalf.

Response: We have amended the Funding Statement to include all grant support and we have provide an amended statement that declares all funding or sources of support and includes “There was no additional external funding received for this study.” in the revised cover letter.

3. Thank you for stating the following financial disclosure:

“This work was financially supported by the Xiamen Major Science and Technology Program (Milestone Project, 3502Z20254028, Huiming Ye), the Medical and Industrial Integration Guidance Project of Xiamen (3502Z20244ZD2043, Min Zhu), the Fujian Provincial Natural Science Foundation of China (2023J05266, Jiali Cao). “

Please state what role the funders took in the study. If the funders had no role, please state: "The funders had no role in study design, data collection and analysis, decision to publish, or preparation of the manuscript."

If this statement is not correct you must amend it as needed.

Please include this amended Role of Funder statement in your cover letter; we will change the online submission form on your behalf.

Response: We have stated "The funders had no role in study design, data collection and analysis, decision to publish, or preparation of the manuscript." in the revised cover letter.

4.In the online submission form you indicate that your data is not available for proprietary reasons and have provided a contact point for accessing this data. Please note that your current contact point is a co-author on this manuscript. According to our Data Policy, the contact point must not be an author on the manuscript and must be an institutional contact, ideally not an individual. Please revise your data statement to a non-author institutional point of contact, such as a data access or ethics committee, and send this to us via return email. Please also include contact information for the third party organization, and please include the full citation of where the data can be found.

Response: All de-identified raw data generated and analyzed in this study have been uploaded as supplementary files alongside the revised manuscript. We have revised the “Availability of data” section accordingly.

5. Please be informed that funding information should not appear in the Acknowledgments section or other areas of your manuscript. We will only publish funding information present in the Funding Statement section of the online submission form. Please remove any funding-related text from the manuscript.

Response: We have removed funding-related text from the manuscript.

6. Thank you for stating the following in the Financial Disclosure:

“This work was financially supported by the Xiamen Major Science and Technology Program (Milestone Project, 3502Z20254028, Huiming Ye), the Medical and Industrial Integration Guidance Project of Xiamen (3502Z20244ZD2043, Min Zhu), the Fujian Provincial Natural Science Foundation of China (2023J05266, Jiali Cao).”

We note that one or more of the authors are employed by a commercial company: Xiamen Innodx Biotech Co., Ltd

a. Please provide an amended Funding Statement declaring this commercial affiliation, as well as a statement regarding the Role of Funders in your study. If the funding organization did not play a role in the study design, data collection and analysis, decision to publish, or preparation of the manuscript and only provided financial support in the form of authors' salaries and/or research materials, please review your statements relating to the author contributions, and ensure you have specifically and accurately indicated the role(s) that these authors had in your study. You can update author roles in the Author Contributions section of the online submission form.

Please also include the following statement within your amended Funding Statement.

“The funder provided support in the form of salaries for authors [insert relevant initials], but did not have any additional role in the study design, data collection and analysis, decision to publish, or preparation of the manuscript. The specific roles of these authors are articulated in the ‘author contributions’ section.”

If your commercial affiliation did play a role in your study, please state and explain this role within your updated Funding Statement.

Response: We have declared LL’s commercial affiliation and salary support and clarified that the company had no additional role in the study.

b. Please also provide an updated Competing Interests Statement declaring this commercial affiliation along with any other relevant declarations relating to employment, consultancy, patents, products in development, or marketed products, etc.

Within your Competing Interests Statement, please confirm that this commercial affiliation does not alter your adherence to all PLOS ONE policies on sharing data and materials by including the following statement: "This does not alter our adherence to PLOS ONE policies on sharing data and materials.” (as detailed online in our guide for authors http://journals.plos.org/plosone/s/competing-interests) . If this adherence statement is not accurate and there are restrictions on sharing of data and/or materials, please state these. Please note that we cannot proceed with consideration of your article until this information has been declared.

Please include both an updated Funding Statement and Competing Interests Statement in your cover letter. We will change the online submission form on your behalf.

Response: We have submitted an updated Competing Interests Statement. Lihua Li is employed by Xiamen Innodx Biotech Co., Ltd.. This does not alter our adherence to PLOS ONE policies on sharing data and materials. The other authors declare no competing interests.

7. Your ethics statement should only appear in the Methods section of your manuscript. If your ethics statement is written in any section besides the Methods, please delete it from any other section.

Response: We deleted the duplicate ethics statement from the declarations section.

8. Please ensure that you refer to Figure 2 in your text as, if accepted, production will need this reference to link the reader to the figure.

Response: We added explicit references to Figure 2.

9. We note you have included a table to which you do not refer in the text of your manuscript. Please ensure that you refer to Table 5 in your text; if accepted, production will need this reference to link the reader to the Table

Response: We added explicit references to Table 5.

10. If the reviewer comments include a recommendation to cite specific previously published works, please review and evaluate these publications to determine whether they are relevant and should be cited. There is no requirement to cite these works unless the editor has indicated otherwise.

Response: No new citations were required by the reviewers. We reviewed the reference list, removed the duplicated CNAS-GL037 citation.

Reviewers' comments:

Reviewer's Responses to Questions

Comments to the Author

1. Is the manuscript technically sound, and do the data support the conclusions?

The manuscript must describe a technically sound piece of scientific research with data that supports the conclusions. Experiments must have been conducted rigorously, with appropriate controls, replication, and sample sizes. The conclusions must be drawn appropriately based on the data presented.

Reviewer #1: Yes

Reviewer #2: Yes

2. Has the statistical analysis been performed appropriately and rigorously?

Reviewer #1: I Don't Know

Reviewer #2: I Don't Know

3. Have the authors made all data underlying the findings in their manuscript fully available?

The PLOS Data policy requires authors to make all data underlying the findings described in their manuscript fully available without restriction, with rare exception (please refer to the Data Availability Statement in the manuscript PDF file). The data should be provided as part of the manuscript or its supporting information, or deposited to a public repository. For example, in addition to summary statistics, the data points behind means, medians and variance measures should be available. If there are restrictions on publicly sharing data—e.g. participant privacy or use of data from a third party—those must be specified.

Reviewer #1: Yes

Reviewer #2: Yes

4. Is the manuscript presented in an intelligible fashion and written in standard English?

PLOS ONE does not copyedit accepted manuscripts, so the language in submitted articles must be clear, correct, and unambiguous. Any typographical or grammatical errors should be corrected at revision, so please note any specific errors here.

Reviewer #1: Yes

Reviewer #2: Yes

5. Review Comments to the Author

Please use the space provided to explain your answers to the questions above. You may also include additional comments for the author, including concerns about dual publication, research ethics, or publication ethics. (Please upload your review as an attachment if it exceeds 20,000 characters)

Reviewer #1: Please resolve the age discrepancy: Abstract (20–44) vs. Methods (22–44). Which is correct?

Response: Thank you for identifying this inconsistency. The correct participant age range is 20–45 years. We have revised to make the age range consistent throughout the manuscript.

Why was the accuracy acceptance criterion set at 12.5% (very loose)? Can you provide bias data against a stricter cut-off (e.g., < 7.5% or < 10%)?

Response: The 12.5% criterion was based on the WS/T 492-2016 verification framework, in which bias is evaluated against ≤1/2 of the total allowable error. We agree that a stricter interpretation is useful. The observed absolute biases were 4.20% and 3.26%, respectively; therefore, the assay meets not only the prespecified <12.5% criterion, but also stricter <10%, <7.5%, and <5% thresholds at both tested levels.

How do you explain the slope of 1.19 between Innodx and Roche? Does this mean the two assays are not interchangeable?

Response: We agree. The Innodx–Roche slope of 1.19 and intercept of -3.97 indicate proportional and constant method-specific differences despite a small mean Bland–Altman bias. Therefore, the two assays should not be considered interchangeable for individual patient monitoring or for direct comparison across studies without method-specific consideration.

Can you provide a Bland-Altman plot comparing Innodx and Roche directly (not only each vs. LC-MS/MS)?

Response: The direct Innodx versus Roche Bland–Altman plot is included in Figure 2A.

Reviewer #2: Discuss that the assay meets even the most stringent criteria (<5% at both levels tested)

Response: We agree and have added this point. The observed absolute biases were 4.20% and 3.26%, both below 5%.

The authors MUST address:

Clinical implications: At clinically relevant thresholds:

At 20 ng/mL (deficiency cutoff): Innodx ≈ 20 × 1.19 - 3.97 ≈ 19.8 ng/mL vs Roche at 20 ng/mL → minimal difference

At 30 ng/mL (sufficiency cutoff): Innodx ≈ 30 × 1.19 - 3.97 ≈ 31.7 ng/mL vs Roche at 30 ng/mL → ~1.7 ng/mL higher

At 10 ng/mL (severe deficiency): Innodx ≈ 10 × 1.19 - 3.97 ≈ 7.9 ng/mL vs Roche at 10 ng/mL → clinically meaningful difference

The two assays are not interchangeable for individual patient monitoring or across studies using different assays.

Response: We agree. The revised Discussion now states that the assays should not be used interchangeably for individual longitudinal monitoring or direct cross-study comparison.

Sample Size Limitations

Method comparison used n=100, which is acceptable for pilot studies but relatively small for Passing-Bablok regression.

Response: We agree. We expanded the Limitations section to state that the n=100 method-comparison subset is acceptable for preliminary evaluation but relatively limited for precise Passing–Bablok estimates.

Serum-Plasma Comparison

The regression equation shows y = 0.95 + 0.64x (R=0.982), suggesting substantial proportional bias (slope = 0.64).

Question: Is this equation correct? A slope of 0.64 for serum vs. plasma is quite low and suggests:

Plasma values are about 64% of serum values? OR

This is a typo (should be y = 0.95 + 0.96x)?

Response: This was a typographical error. The correct equation is y = 0.64 + 0.95x (r=0.982).

The table 7 is poorly formatted and difficult to interpret.

Response: We thought that the reviewer mean Table 6 is poorly formatted. We have reformatted Table 6 to simplify its layout and improve readability.

Abstract line 36: Uses "≥30≥30" but line 35-36 says "sufficient (> 30 ng/mL)"—be consistent

Response: We standardized the category definitions throughout the manuscript to avoid overlap: deficiency <20 ng/mL, insufficiency ≥20, <30 ng/mL, and sufficiency ≥30 ng/mL.

Reference formatting: References 11, 12, 16 appear duplicated (CNAS-GL037 appears twice)

Response: Thank you. The duplicated CNAS-GL037 entry was removed, and the subsequent references were renumbered.

Line 88: "fat-soluble steroid hormone" → technically vitamin D is a secosteroid, not a steroid

Response: We revised this phrase.

Line 84-85: The Roche assay and Mindray assay both use pH-shift methods—this is correctly stated

Typo: "rheumatoid fator" in Table 4 → should be "rheumatoid factor"

Response: Thank you. The typographical error has been corrected.

6. PLOS authors have the option to publish the peer review history of their article (what does this mean?). If published, this will include your full peer review and any attached files.

If you choose “no”, your identity will remain anonymous but your review may still be made public

Response: We choose “no”.

We believe that these revisions have addressed all editorial and reviewer concerns and have improved the clarity and rigor of the manuscript.

Sincerely,

The Authors
