# Supplementary material for: Performance evaluation of a direct chemiluminescence assay for 25-hydroxyvitamin D and its application in assessing vitamin D status among reproductive-age women in southeastern China
Source: PLoS One. 2026 Sep 15;21(9):e0356809. doi: 10.1371/journal.pone.0356809 (PMC13577506; doi:10.1371/journal.pone.0356809)
Supplement: Peer review history

# Peer review: Decision Letter 0

**DOI:** 10.1371/journal.pone.0356809.r001

**Authors:** Sanaz Alaeejahromi

7 Jul 2026

Dear Dr. Huiming Ye

Thank you for submitting your manuscript to PLOS ONE. After careful consideration, we feel that it has merit but does not fully meet PLOS ONE’s publication criteria as it currently stands. Therefore, we invite you to submit a revised version of the manuscript that addresses the points raised during the review process.

Please submit your revised manuscript by Aug 21 2026 11:59PM. If you will need more time than this to complete your revisions, please reply to this message or contact the journal office at plosone@plos.org. When you're ready to submit your revision, log on to https://www.editorialmanager.com/pone/ and select the 'Submissions Needing Revision' folder to locate your manuscript file.

- A letter that responds to each point raised by the academic editor and reviewer(s). You should upload this letter as a separate file labeled 'Response to Reviewers'.
- A marked-up copy of your manuscript that highlights changes made to the original version. You should upload this as a separate file labeled 'Revised Manuscript with Track Changes'.
- An unmarked version of your revised paper without tracked changes. You should upload this as a separate file labeled 'Manuscript'.

If you would like to make changes to your financial disclosure, please include your updated statement in your cover letter. Guidelines for resubmitting your figure files are available below the reviewer comments at the end of this letter.

If applicable, we recommend that you deposit your laboratory protocols in protocols.io to enhance the reproducibility of your results. Protocols.io assigns your protocol its own identifier (DOI) so that it can be cited independently in the future. For instructions see: https://journals.plos.org/plosone/s/submission-guidelines#loc-laboratory-protocols. Additionally, PLOS ONE offers an option for publishing peer-reviewed Lab Protocol articles, which describe protocols hosted on protocols.io. Read more information on sharing protocols at https://plos.org/protocols?utm_medium=editorial-email&utm_source=authorletters&utm_campaign=protocols.

As the corresponding author, your ORCID iD is verified in the submission system and will appear in the published article. PLOS supports the use of ORCID, and we encourage all coauthors to register for an ORCID iD and use it as well. Please encourage your coauthors to verify their ORCID iD within the submission system before final acceptance, as unverified ORCID iDs will not appear in the published article. *Only*  the individual author can complete the verification step; PLOS staff *cannot*  verify ORCID iDs on behalf of authors.

We look forward to receiving your revised manuscript.

Kind regards,

Sanaz Alaeejahromi

Academic Editor

PLOS One

Journal Requirements:

When submitting your revision, we need you to address these additional requirements.

1. Please ensure that your manuscript meets PLOS ONE's style requirements, including those for file naming. The PLOS ONE style templates can be found at

https://journals.plos.org/plosone/s/file?id=wjVg/PLOSOne_formatting_sample_main_body.pdf and

https://journals.plos.org/plosone/s/file?id=ba62/PLOSOne_formatting_sample_title_authors_affiliations.pdf.

2. Thank you for stating in your Funding Statement:

“This work was financially supported by the Xiamen Major Science and Technology Program (Milestone Project, 3502Z20254028, Huiming Ye), the Medical and Industrial Integration Guidance Project of Xiamen (3502Z20244ZD2043, Min Zhu), the Fujian Provincial Natural Science Foundation of China (2023J05266, Jiali Cao). “

Please provide an amended statement that declares *all* the funding or sources of support (whether external or internal to your organization) received during this study, as detailed online in our guide for authors at http://journals.plos.org/plosone/s/submit-now.  Please also include the statement “There was no additional external funding received for this study.” in your updated Funding Statement.

Please include your amended Funding Statement within your cover letter. We will change the online submission form on your behalf.

3. Thank you for stating the following financial disclosure:

“This work was financially supported by the Xiamen Major Science and Technology Program (Milestone Project, 3502Z20254028, Huiming Ye), the Medical and Industrial Integration Guidance Project of Xiamen (3502Z20244ZD2043, Min Zhu), the Fujian Provincial Natural Science Foundation of China (2023J05266, Jiali Cao). “

Please state what role the funders took in the study.  If the funders had no role, please state: "The funders had no role in study design, data collection and analysis, decision to publish, or preparation of the manuscript."

If this statement is not correct you must amend it as needed.

Please include this amended Role of Funder statement in your cover letter; we will change the online submission form on your behalf.

4.In the online submission form you indicate that your data is not available for proprietary reasons and have provided a contact point for accessing this data. Please note that your current contact point is a co-author on this manuscript. According to our Data Policy, the contact point must not be an author on the manuscript and must be an institutional contact, ideally not an individual. Please revise your data statement to a non-author institutional point of contact, such as a data access or ethics committee, and send this to us via return email. Please also include contact information for the third party organization, and please include the full citation of where the data can be found.

5. Please be informed that funding information should not appear in the Acknowledgments section or other areas of your manuscript. We will only publish funding information present in the Funding Statement section of the online submission form. Please remove any funding-related text from the manuscript.

6. Thank you for stating the following in the Financial Disclosure:

“This work was financially supported by the Xiamen Major Science and Technology Program (Milestone Project, 3502Z20254028, Huiming Ye), the Medical and Industrial Integration Guidance Project of Xiamen (3502Z20244ZD2043, Min Zhu), the Fujian Provincial Natural Science Foundation of China (2023J05266, Jiali Cao).”

We note that one or more of the authors are employed by a commercial company:  Xiamen Innodx Biotech Co., Ltd

a.        Please provide an amended Funding Statement declaring this commercial affiliation, as well as a statement regarding the Role of Funders in your study. If the funding organization did not play a role in the study design, data collection and analysis, decision to publish, or preparation of the manuscript and only provided financial support in the form of authors' salaries and/or research materials, please review your statements relating to the author contributions, and ensure you have specifically and accurately indicated the role(s) that these authors had in your study. You can update author roles in the Author Contributions section of the online submission form.

Please also include the following statement within your amended Funding Statement.

“The funder provided support in the form of salaries for authors [insert relevant initials], but did not have any additional role in the study design, data collection and analysis, decision to publish, or preparation of the manuscript. The specific roles of these authors are articulated in the ‘author contributions’ section.”

If your commercial affiliation did play a role in your study, please state and explain this role within your updated Funding Statement.

b. Please also provide an updated Competing Interests Statement declaring this commercial affiliation along with any other relevant declarations relating to employment, consultancy, patents, products in development, or marketed products, etc.

Within your Competing Interests Statement, please confirm that this commercial affiliation does not alter your adherence to all PLOS ONE policies on sharing data and materials by including the following statement: "This does not alter our adherence to  PLOS ONE policies on sharing data and materials.” (as detailed online in our guide for authors http://journals.plos.org/plosone/s/competing-interests) . If this adherence statement is not accurate and  there are restrictions on sharing of data and/or materials, please state these. Please note that we cannot proceed with consideration of your article until this information has been declared.

Please include both an updated Funding Statement and Competing Interests Statement in your cover letter. We will change the online submission form on your behalf.

7. Your ethics statement should only appear in the Methods section of your manuscript. If your ethics statement is written in any section besides the Methods, please delete it from any other section.

8. Please ensure that you refer to Figure 2 in your text as, if accepted, production will need this reference to link the reader to the figure.

9. We note you have included a table to which you do not refer in the text of your manuscript. Please ensure that you refer to Table 5 in your text; if accepted, production will need this reference to link the reader to the Table

10. If the reviewer comments include a recommendation to cite specific previously published works, please review and evaluate these publications to determine whether they are relevant and should be cited. There is no requirement to cite these works unless the editor has indicated otherwise.

Reviewers' comments:

Reviewer's Responses to Questions

**Comments to the Author**

1. Is the manuscript technically sound, and do the data support the conclusions?

Reviewer #1: Yes

Reviewer #2: Yes

**********

2. Has the statistical analysis been performed appropriately and rigorously?

Reviewer #1: I Don't Know

Reviewer #2: I Don't Know

**********

3. Have the authors made all data underlying the findings in their manuscript fully available?

The PLOS Data policy

Reviewer #1: Yes

Reviewer #2: Yes

**********

4. Is the manuscript presented in an intelligible fashion and written in standard English?

Reviewer #1: Yes

Reviewer #2: Yes

**********

Reviewer #1: Please resolve the age discrepancy: Abstract (20–44) vs. Methods (22–44). Which is correct?

Why was the accuracy acceptance criterion set at 12.5% (very loose)? Can you provide bias data against a stricter cut-off (e.g., < 7.5% or < 10%)?

How do you explain the slope of 1.19 between Innodx and Roche? Does this mean the two assays are not interchangeable?

Can you provide a Bland-Altman plot comparing Innodx and Roche directly (not only each vs. LC-MS/MS)?

Reviewer #2: Discuss that the assay meets even the most stringent criteria (<5% at both levels tested)

The authors MUST address:

Clinical implications: At clinically relevant thresholds:

At 20 ng/mL (deficiency cutoff): Innodx ≈ 20 × 1.19 - 3.97 ≈ 19.8 ng/mL vs Roche at 20 ng/mL → minimal difference

At 30 ng/mL (sufficiency cutoff): Innodx ≈ 30 × 1.19 - 3.97 ≈ 31.7 ng/mL vs Roche at 30 ng/mL → ~1.7 ng/mL higher

At 10 ng/mL (severe deficiency): Innodx ≈ 10 × 1.19 - 3.97 ≈ 7.9 ng/mL vs Roche at 10 ng/mL → clinically meaningful difference

The two assays are not interchangeable for individual patient monitoring or across studies using different assays.

Sample Size Limitations

Method comparison used n=100, which is acceptable for pilot studies but relatively small for Passing-Bablok regression.

Serum-Plasma Comparison

The regression equation shows y = 0.95 + 0.64x (R=0.982), suggesting substantial proportional bias (slope = 0.64).

Question: Is this equation correct? A slope of 0.64 for serum vs. plasma is quite low and suggests:

Plasma values are about 64% of serum values? OR

This is a typo (should be y = 0.95 + 0.96x)?

The table 7 is poorly formatted and difficult to interpret.

Abstract line 36: Uses "≥30≥30" but line 35-36 says "sufficient (> 30 ng/mL)"—be consistent

Reference formatting: References 11, 12, 16 appear duplicated (CNAS-GL037 appears twice)

Line 88: "fat-soluble steroid hormone" → technically vitamin D is a secosteroid, not a steroid

Line 84-85: The Roche assay and Mindray assay both use pH-shift methods—this is correctly stated

Typo: "rheumatoid fator" in Table 4 → should be "rheumatoid factor"

**********

what does this mean?). If published, this will include your full peer review and any attached files.

To ensure your figures meet our technical requirements, please review our figure guidelines: https://journals.plos.org/plosone/s/figures

You may also use PLOS’s free figure tool, NAAS, to help you prepare publication quality figures: https://journals.plos.org/plosone/s/figures#loc-tools-for-figure-preparation.

NAAS will assess whether your figures meet our technical requirements by comparing each figure against our figure specifications.

# Author comment: Author response to Decision Letter 1

**DOI:** 10.1371/journal.pone.0356809.r002

13 Jul 2026

Reviewer #1: Please resolve the age discrepancy: Abstract (20–44) vs. Methods (22–44). Which is correct?

Response: Thank you for identifying this inconsistency. The correct participant age range is 20–45 years. We have revised to make the age range consistent throughout the manuscript.

Why was the accuracy acceptance criterion set at 12.5% (very loose)? Can you provide bias data against a stricter cut-off (e.g., < 7.5% or < 10%)?

Response: The 12.5% criterion was based on the WS/T 492-2016 verification framework, in which bias is evaluated against ≤1/2 of the total allowable error. We agree that a stricter interpretation is useful. The observed absolute biases were 4.20% and 3.26%, respectively; therefore, the assay meets not only the prespecified <12.5% criterion, but also stricter <10%, <7.5%, and <5% thresholds at both tested levels.

How do you explain the slope of 1.19 between Innodx and Roche? Does this mean the two assays are not interchangeable?

Response: We agree. The Innodx–Roche slope of 1.19 and intercept of -3.97 indicate proportional and constant method-specific differences despite a small mean Bland–Altman bias. Therefore, the two assays should not be considered interchangeable for individual patient monitoring or for direct comparison across studies without method-specific consideration.

Can you provide a Bland-Altman plot comparing Innodx and Roche directly (not only each vs. LC-MS/MS)?

Response: The direct Innodx versus Roche Bland–Altman plot is included in Figure 2A.

Reviewer #2: Discuss that the assay meets even the most stringent criteria (<5% at both levels tested)

Response: We agree and have added this point. The observed absolute biases were 4.20% and 3.26%, both below 5%.

The authors MUST address:

Clinical implications: At clinically relevant thresholds:

At 20 ng/mL (deficiency cutoff): Innodx ≈ 20 × 1.19 - 3.97 ≈ 19.8 ng/mL vs Roche at 20 ng/mL → minimal difference

At 30 ng/mL (sufficiency cutoff): Innodx ≈ 30 × 1.19 - 3.97 ≈ 31.7 ng/mL vs Roche at 30 ng/mL → ~1.7 ng/mL higher

At 10 ng/mL (severe deficiency): Innodx ≈ 10 × 1.19 - 3.97 ≈ 7.9 ng/mL vs Roche at 10 ng/mL → clinically meaningful difference

The two assays are not interchangeable for individual patient monitoring or across studies using different assays.

Response: We agree. The revised Discussion now states that the assays should not be used interchangeably for individual longitudinal monitoring or direct cross-study comparison.

Sample Size Limitations

Method comparison used n=100, which is acceptable for pilot studies but relatively small for Passing-Bablok regression.

Response: We agree. We expanded the Limitations section to state that the n=100 method-comparison subset is acceptable for preliminary evaluation but relatively limited for precise Passing–Bablok estimates.

Serum-Plasma Comparison

The regression equation shows y = 0.95 + 0.64x (R=0.982), suggesting substantial proportional bias (slope = 0.64).

Question: Is this equation correct? A slope of 0.64 for serum vs. plasma is quite low and suggests:

Plasma values are about 64% of serum values? OR

This is a typo (should be y = 0.95 + 0.96x)?

Response: This was a typographical error. The correct equation is y = 0.64 + 0.95x (r=0.982).

The table 7 is poorly formatted and difficult to interpret.

Response: We thought that the reviewer mean Table 6 is poorly formatted. We have reformatted Table 6 to simplify its layout and improve readability.

Abstract line 36: Uses "≥30≥30" but line 35-36 says "sufficient (> 30 ng/mL)"—be consistent

Response: We standardized the category definitions throughout the manuscript to avoid overlap: deficiency <20 ng/mL, insufficiency ≥20, <30 ng/mL, and sufficiency ≥30 ng/mL.

Reference formatting: References 11, 12, 16 appear duplicated (CNAS-GL037 appears twice)

Response: Thank you. The duplicated CNAS-GL037 entry was removed, and the subsequent references were renumbered.

Line 88: "fat-soluble steroid hormone" → technically vitamin D is a secosteroid, not a steroid

Response: We revised this phrase.

Line 84-85: The Roche assay and Mindray assay both use pH-shift methods—this is correctly stated

Typo: "rheumatoid fator" in Table 4 → should be "rheumatoid factor"

Response: Thank you. The typographical error has been corrected.

Attachment Submitted filename: Response to Reviewers.docx

# Peer review: Decision Letter 1

**DOI:** 10.1371/journal.pone.0356809.r003

**Authors:** Sanaz Alaeejahromi

10 Aug 2026

Performance evaluation of a direct chemiluminescence assay for 25-hydroxyvitamin D and its application in assessing vitamin D status among reproductive-age women in southeastern China

PONE-D-26-17612R1

Dear Dr. Huiming Ye

We’re pleased to inform you that your manuscript has been judged scientifically suitable for publication and will be formally accepted for publication once it meets all outstanding technical requirements.

Within one week, you’ll receive an e-mail detailing the required amendments. When these have been addressed, you’ll receive a formal acceptance letter and your manuscript will be scheduled for publication.

An invoice will be generated when your article is formally accepted. Please note, if your institution has a publishing partnership with PLOS and your article meets the relevant criteria, all or part of your publication costs will be covered. Please make sure your user information is up-to-date by logging into Editorial Manager at Editorial Manager® and clicking the ‘Update My Information' link at the top of the page. For questions related to billing, please contact billing support.

If your institution or institutions have a press office, please notify them about your upcoming paper to help maximize its impact. If they’ll be preparing press materials, please inform our press team as soon as possible -- no later than 48 hours after receiving the formal acceptance. Your manuscript will remain under strict press embargo until 2 pm Eastern Time on the date of publication. For more information, please contact onepress@plos.org.

Kind regards,

Sanaz Alaeejahromi

Academic Editor

PLOS One

Reviewers' comments:

Reviewer's Responses to Questions

**Comments to the Author**

Reviewer #1: All comments have been addressed

Reviewer #3: All comments have been addressed

**********

2. Is the manuscript technically sound, and do the data support the conclusions?

Reviewer #1: Yes

Reviewer #3: Yes

**********

3. Has the statistical analysis been performed appropriately and rigorously?

Reviewer #1: Yes

Reviewer #3: I Don't Know

**********

4. Have the authors made all data underlying the findings in their manuscript fully available?

The PLOS Data policy

Reviewer #1: Yes

Reviewer #3: Yes

**********

5. Is the manuscript presented in an intelligible fashion and written in standard English?

Reviewer #1: Yes

Reviewer #3: Yes

**********

Reviewer #1: (No Response)

Reviewer #3: would like to thank the authors for the careful and thorough revision. All previous concerns have been addressed with clarity and rigor. The manuscript is now much improved, and I have no further requests. It has been a pleasure reviewing this work, and I congratulate the author on an excellent study.

# Editor report: Acceptance letter

**DOI:** 10.1371/journal.pone.0356809.r004

**Authors:** Sanaz Alaeejahromi

PONE-D-26-17612R1

PLOS One

Dear Dr. Ye,

I'm pleased to inform you that your manuscript has been deemed suitable for publication in PLOS One. Congratulations! Your manuscript is now being handed over to our production team.

At this stage, our production department will prepare your paper for publication. This includes ensuring the following:

* All references, tables, and figures are properly cited

* All relevant supporting information is included in the manuscript submission,

* There are no issues that prevent the paper from being properly typeset

You will receive further instructions from the production team, including instructions on how to review your proof when it is ready. Please keep in mind that we are working through a large volume of accepted articles, so please give us a few days to review your paper and let you know the next and final steps.

Lastly, if your institution or institutions have a press office, please let them know about your upcoming paper now to help maximize its impact. If they'll be preparing press materials, please inform our press team within the next 48 hours. Your manuscript will remain under strict press embargo until 2 pm Eastern Time on the date of publication. For more information, please contact onepress@plos.org.

You will receive an invoice from PLOS for your publication fee after your manuscript has reached the completed accept phase. If you receive an email requesting payment before acceptance or for any other service, this may be a phishing scheme. Learn how to identify phishing emails and protect your accounts at https://explore.plos.org/phishing.

If we can help with anything else, please email us at customercare@plos.org.

Thank you for submitting your work to PLOS One and supporting open access.

Kind regards,

PLOS One Editorial Office Staff

on behalf of

Dr. Sanaz Alaeejahromi

Academic Editor

PLOS One
